# Acetated Tincture of Cimicifuga

**Published:** 1859-05

**Authors:** 


ACETATED TINCTURE OF CIMICIFUGA.—In our January number we gave a formula for the preparation of this remedy, and we have received a note from Dr. Koehler, in which he states that instead of using dilute acetic acid, it should be employed in its pure state.

